# Protective effect of docosahexaenoic acid on lipotoxicity‐mediated cell death in Schwann cells: Implication of PI3K/AKT and mTORC2 pathways

**DOI:** 10.1002/brb3.1123

**Published:** 2018-09-28

**Authors:** Magda Descorbeth, Karen Figueroa, Miguel Serrano‐Illán, Marino De León

**Affiliations:** ^1^ Center for Health Disparities and Molecular Medicine and Department of Basic Sciences Loma Linda University School of Medicine Loma Linda California

**Keywords:** AKT phosphorylation, docosahexaenoic acid, PA‐induced lipotoxicity, primary cultured Schwann cells

## Abstract

**Background and Aim:**

Docosahexaenoic acid (DHA) exhibits neuroprotective properties and has been shown to preserve nerve cells following trauma and ischemic injury. Recently, we showed that DHA pretreatment improved locomotion and reduced neuropathic pain after acute spinal cord injury in adult rats. These improvements were associated with an increase in the levels of AKT in spinal cord injury neurons. In this study, we investigate the implication of PI3K/AKT and mTOR pathway in DHA‐mediated protection of primary cultured Schwann cells (pSC) undergoing palmitic acid‐induced lipotoxicity (PA‐LTx).

**Methods:**

Primary cultured Schwann cells were treated with PA (PA:BSA, 2:1) in the presence or absence of DHA (1–200 µM) for 24–48 hr. Cell viability was determined by crystal violet staining and nuclear morphology was examined using Hoechst staining.

**Results:**

We found that pSC cultures exposed to palmitic acid (PA) overload showed chromatin condensation, a decrease in cell viability and an inhibition of AKT phosphorylation in a time‐dependent manner. Next, pSC exposed to PA overload were treated with DHA. The data show that co‐treatment with DHA inhibited the loss of cell viability and apoptosis caused by PA. Moreover, treatment with DHA inhibited chromatin condensation, significantly stimulated p‐AKT phosphorylation under PA‐LTx condition, and DHA alone increased AKT phosphorylation. Additionally, when these pSC cultures were treated with PI3K inhibitors LY294002 and, BKM120 and mTOR inhibitors Torin 1 (mTORC1/mTORC2), but not rapamycin (mTORC1), the protective effects of DHA were not observed.

**Conclusion:**

These findings suggest PI3K/AKT and mTORC2 kinase pathways are involved in the protective function (s) of DHA in PA‐induced Schwann cell death.

## 1 INTRODUCTION

Along with hyperglycemia, hyperlipidemia plays a significant role in insulin resistance and type 2 diabetes‐related complications (Ghosh & Rodrigues, 2006; Ioannidis, 2008; Kusminski, Shetty, Orci, Unger, & Scherer, 2009; Unger, 2008; Wilding, 2007). The cellular lipotoxicity and apoptotic cell death that follows saturated fatty acid overload have been well documented by our group and others in several cell types and tissues (Listenberger et al., 2003; van Herpen & Schrauwen‐Hinderling, 2008). Neurons and Schwann cells that are chronically exposed to high levels of saturated fatty acids like palmitic acid (PA) exhibit an apoptotic cell death that involves a strong ER stress response, mitochondrial and lysosomal dysfunction, and the formation of reactive oxygen species (ROS; Almaguel, Liu, Pacheco, Casiano, & De Leon, 2009; Liu, Montero, Bu, & De Leon, 2015; Padilla, Descorbeth, Almeyda, Payne, & De Leon, 2011; Ulloth, Casiano, & De Leon, 2003).

In contrast to PA, the polyunsaturated fatty acid docosahexaenoic acid (DHA) exhibits neuroprotective properties such as reduced infarct volume and decreased mortality from focal cerebral ischemia (Almaguel et al., 2009; Bazan, 2006; Belayev et al., 2011; Belayev, Khoutorova, Atkins, & Bazan, 2009). For instance, we found that DHA protects nerve growth factor‐differentiated PC12 (NGFDPC12) and rat cortical cells (RCC) from PA‐LTx by stabilizing lysosomal and mitochondrial membrane potential. DHA also reduced apoptosis by inhibiting caspase‐3 activation, and the cleavage of PARP1 and lamin‐B under PA conditions (Almaguel et al., 2009, 2010 ). In a series of studies using an in vivo rat spinal injury model, we recently reported that acute parenteral administration of DHA prior to injury and a diet rich in menhaden fish oil‐derived omega‐3 fatty acids improved the conduction and locomotor function of rats after spinal cord injury (SCI; Figueroa & De Leon, 2014; Figueroa et al., 2012; Figueroa, Cordero, Llan, & De Leon, 2013). These studies show that DHA pretreatment increased the percentage of white matter sparing resulting in axonal preservation. Overall, DHA seems to have therapeutic potential in the treatment and prevention of neurological deficits associated with spinal cord injury and neuropathic pain. Understanding the underlying molecular mechanism (s) by which DHA confers its protection may provide insightful information relevant for the neuropathic pain field.

AKT, also known as protein kinase B (PKB), is a serine/threonine kinase downstream of the phosphatidylinositide 3‐kinases (PI3K). This prominent cellular signaling pathway plays a key role during cell growth and survival. DHA increases phosphatidylserine (PS) in neural cells which results in translocation of AKT from the cytosol to the membrane for phosphorylation and activation after ischemia (Akbar, Calderon, Wen, & Kim, 2005; Guo, Stockert, Akbar, & Kim, 2007). Akbar and Kim (2002) reported that DHA enrichment protected Neuro 2A cells from the apoptotic cell death induced by staurosporine, a protein kinase C inhibitor and that this effect was partially mediated by the PI3K/AKT pathway. Pertinent to the present study, DHA pretreatment also increased the levels of AKT in spinal cord injured rats (Figueroa et al., 2012, 2013 ). Phosphorylated AKT was expressed highly in the nuclear and perinuclear regions of neuron like cells as well as in oligodendrocytes and astrocytes cells (Figueroa et al., 2012).

The mammalian target of rapamycin (mTOR) is an important nutrient sensor that regulates cell growth, proliferation, and survival (Um, D'Alessio, & Thomas, 2006; Wullschleger, Loewith, & Hall, 2006). There are two distinct complexes of mTOR: mTORC1 and mTORC2. mTORC1 is a rapamycin sensitive complex, a nutrient/energy/redox sensor consisting of mTOR (Babaev et al., 2016). In contrast mTORC2, which is rapamycin insensitive (Loewith et al., 2002; Wullschleger et al., 2006) activates AKT through phosphorylation at Ser473 and promotes cell survival (Laplante & Sabatini, 2009). Upon activation, AKT activates mTORC1 via the phosphorylation of TSC1/2 (Kim, Cook, & Chen, 2017; Sancak et al., 2007; Vander Haar, Lee, Bandhakavi, Griffin, & Kim, 2007). The objective of the present study is to determine the involvement of PI3K/AKT and mTOR pathways in DHA‐mediated protection of primary cultured Schwann cells (pSC) against PA‐induced lipotoxicity (PA‐LTx). We report that pSC cultures exposed to a PA overload exhibit lipotoxicity and cell death that is associated with a decrease in AKT phosphorylation. In contrast, treatment with DHA improves cell survival and confers protection through the PI3K/AKT and mTORC2 pathways. Schwann cells (SC) are essential in supporting a healthy myelin sheath for normal nerve conduction and regeneration of injured axons. Dysfunction in these cells results in demyelination and impaired axonal regeneration abnormalities associated with peripheral neuropathies and neuropathic pain (Song et al., 2003).

## 2 MATERIALS AND METHODS

### 2.1 Materials

Rat primary Schwann cells (pSC) were derived from neonate rat sciatic nerves and obtained along with the Schwann cell media (SCM) and Schwann cell growth supplement (SCGS), from ScienCell Research Laboratories™ (Carlsbad, CA). Palmitic acid (PA), DHA, and poly‐L lysine were provided by Sigma‐Aldrich (St. Louis, MO). Fatty acid‐free BSA and LY294002 were purchased from EMD Biosciences (San Diego, CA). MedChem Express provided our NVP‐BKM120 (Princeton, NJ). Annexin V FITC and 7‐amino‐actinomycin D (7AAD) were purchased from BD Biosciences (San Diego, CA) and eBioscience (San Diego, CA) respectively. Finally, we obtained the antibodies to total AKT (mouse, Cat# 2920, http://scicrunch.org/resolver/AB_1147620) and phosphorylated AKT Ser473 (rabbit, Cat# 4060, http://scicrunch.org/resolver/AB_2315049) or Thr308 (rabbit, Cat# 2965, http://scicrunch.org/resolver/AB_2255933), and rapamycin from Cell signaling Technology (Danver, MA), the actin (mouse, Cat# A5441, http://scicrunch.org/resolver/AB_476744) from Sigma‐Aldrich (St Louis, MO) and Torin 1 from Tocris Bioscience (Bristol, UK). All of these antibodies are commercially available see characterization details in Table 1.

**Table 1:** Antibody characterization

Antigen	Description of Immunogen	Source, Host species, Cat. No., Lot No., RRID	MW, WB dilution	Citation
Phosphorylated AKT at Ser473	Synthetic phosphopeptide corresponding to residues around Ser473 of human Akt	Cell signaling, Rabbit, Cat no.4060S, Lot:19, http://scicrunch.org/resolver/AB_2315049	MW:60 kDa, Dilution: 1:500	(Cell Signaling Technology Cat# 4060, http://scicrunch.org/resolver/AB_2315049)
Phosphorylated AKT at Thr308	Synthetic phosphopeptide corresponding to residues around Thr308 of mouse Akt	Cell signaling, Rabbit, Cat no. 2965S, Lot:14, http://scicrunch.org/resolver/AB_2255933	MW: 60 kDa, Dilution: 1:500	(Cell Signaling Technology Cat# 2965, http://scicrunch.org/resolver/AB_2255933)
AKT (pan)	Synthetic peptide at the carboxy‐terminal sequence of human Akt	Cell signaling, Mouse, Cat no.2920S, Lot:3, http://scicrunch.org/resolver/AB_1147620	MW: 60 kDa, Dilution: 1:500	Cell Signaling Technology Cat# 2920, http://scicrunch.org/resolver/AB_1147620)
Actin	Modified β‐cytoplasmic actin N‐terminal peptide, Ac‐Asp‐Asp‐Asp‐Ile‐Ala‐Ala‐Leu‐Val‐Ile‐Asp‐Asn‐Gly‐Ser‐Gly‐Lys, conjugated to KLH	Sigma, Mouse, Cat no.A5441, Lot:026M4780V, http://scicrunch.org/resolver/AB_476744	MW: 42 kDa, Dilution: 1:5,000	(Sigma‐Aldrich Cat# A5441, http://scicrunch.org/resolver/AB_476744)

### 2.2 Cell culture and fatty acid treatment

Rat primary Schwann cells (pSC) were cultured in Schwann cell media containing 1% Schwann cell growth serum, 5% fetal bovine serum (FBS), and 1% penicillin/streptomycin in poly‐l lysine (0.01%) coated flasks. Cells are maintained at 37°C with 5% CO_2_ and the media is changed every three days. For the PA‐LTx experiments, pSC cells are plated in 6‐well plates at a density of 40,000 cells per well and cultured with serum‐containing media. Before 24 hr treatment, cells are starved with serum‐free media (SCM containing 1% SCGS and 1% penicillin/streptomycin). Fatty acid treatments are in serum‐free conditions and cells are treated with the PA:BSA (2:1) complex as described before (Almaguel et al., 2009; Padilla et al., 2011). PA and DHA stock is prepared in 100% ethanol at a concentration of 300 mM. Serum‐free media containing 150 µM fatty acid‐free BSA (1% BSA) is prepared and warmed up to 37°C to help to completely disassociate of PA. We added PA stock to the media while vortexing to reach a final concentration of 300 µM (PA:BSA, 2:1). This preparation provides low nanomolar concentration of unbound PA in the medium throughout the course of the experiment (Almaguel et al., 2009). For the control condition, we added 0.01% ethanol to the culture media (control or PA) and then placed it in a water bath at 37°C for 30 min before use. For some groups, we added DHA directly to the treatment media. Finally, we treated the cells with PA, DHA, or PA + DHA for up to 48 hr as needed for the different experiments. DHA concentrations used ranged from 1 µM to 200 µM (DHA:BSA, 1:150 to 4:3). This should result in an unbound DHA concentration in the nanomolar range. We visualized cell morphology on an Olympus IX70 microscope equipped with Hoffman modulation contrast (Olympus American) and a digital spot Imagin system (Diagnostic Instruments, Sterling Heights, MI) as described before (Basu et al., 2016).

### 2.3 Cell viability assay

After PA/DHA treatment, cells were fixed with 4% formaldehyde in PBS for 30 min at room temperature in 6‐well plates. The pSC cells were washed twice with distilled water and allowed to dry completely. Once dry, 1 ml/well of crystal violet dye (Accustain®; Sigma‐Aldrich) was added for 30 min at room temperature. Cells were again washed with distilled water and allowed to dry. Subsequently, bound crystal violet was dissolved with 1 ml/well of 10% acetic acid solution and transferred to a 96‐well plate. Absorbance was read on an µQuant microplate reader using KC Junior software at 570 nM (Bio‐Tek Instruments, Winooski, VT).

### 2.4 Nuclear morphology analysis

Nuclear staining was analyzed with Hoechst 33,342 dye to further assess chromatin condensation in pSC cells. Hoechst 33,342 dye (10 µg/ml) was added to the cultures and incubated for 10 min at 37°C with 5% CO_2_. Nuclear morphology was analyzed using an Olympus fluorescent microscope (excitation/emission wavelength of 365/420 nm) equipped with a SPOT RT3 camera (Diagnostic Instruments). Cells were determined to be apoptotic when they demonstrated apoptotic bodies and increased chromatin condensation.

### 2.5 Western blot analysis

We determined protein expression by Western blots as previously described (Padilla et al., 2011). We separated the extracted proteins from pSC by SDS‐PAGE electrophoresis and then transferred them to a nitrocellulose membrane. Membranes were blocked with Odyssey Blocking Buffer (Li‐Cor Biosciences, Lincoln, NE) at room temperature for 1 hr and then incubated at 4°C overnight with a specific antibody against phospho‐AKT (Ser473), phospho‐AKT (Thr308), or total AKT diluted in PBS with 0.05% Tween 20 (PBST). Subsequently, membranes were washed with PBST and incubated with goat anti‐rabbit IRDye‐800 (Li‐Cor Biosciences) for 1 hr in the dark and at room temperature. After the final wash, we detected the signal with the Odyssey Infrared imager system (Li‐Cor Biosciences). Membranes were then re‐probed with β‐actin (clone AC‐15; Sigma‐Aldrich) and goat anti‐mouse IRDye‐680RD (Li‐Cor Biosciences) to verify loading. Finally, we quantified the protein using Licor Image Studio Software (http://scicrunch.org/resolver/SCR_015795).

### 2.6 Statistical analysis

All experiments were repeated independently at least three times. The values shown are means ± *SEM*. Statistical analysis and compilation graphs were made with GraphPad Prism (http://scicrunch.org/resolver/SCR_002798; San Diego, CA). Groups were compared using independent samples *t* tests or one‐way ANOVA with Bonferroni’s multiple comparison post hoc test. We accepted statistical significance when *p* < 0.05.

## 3 RESULTS

### 3.1 Palmitic acid and PI3K inhibitors decreased cell viability, induced apoptosis and AKT phosphorylation in primary cultured Schwann cells

**Figure 1:**
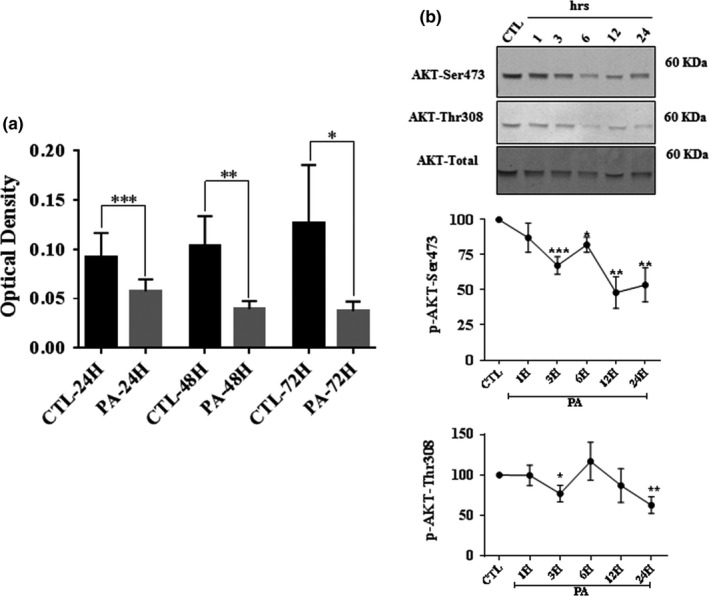
PA‐LTx induced apoptosis and a decrease in AKT phosphorylation in primary Schwann cells (pSC). pSC were treated with BSA alone (control), PA:BSA (2:1) for 24–48 hr. (a) Cell viability was assessed by crystal violet assay. (b) The effect of PA:BSA (2:1) on AKT phosphorylation was examined by Western blot. The pSC cell lysates from control cultures (CTL) exposed to BSA alone and cultures exposed to PA:BSA (2:1) at 1, 3, 6, 12, and 24 hr (hrs.) were prepared and subjected to specific antibodies against AKTp‐Ser473, AKTp‐Thr308 and to total AKT. The blots were then analyzed using the Li‐Cor Odyssey system. A representative Western blot is shown above each bar graph. The data represent mean ± *SE* of at least four independent experiments. *p < 0.05, **p < 0.01, ***p < 0.001 shown above the lines when compared between two linked groups

In agreement with our previous observations (Padilla et al., 2011), when pSC were exposed to PA (PA:BSA, 2:1) overload, Schwann cells viability decreased in a time‐dependent manner (Figure 1a). PA led to a significant decrease in cell viability: 38% (24 hr), 62% (48 hr), and 70% (72 hr) compared to control (BSA alone). The mean of blanked OD reading for each control was 0.092 ± 0.024 at 24 hr, 0.103 ± 0.031 at 48 hr, and 0.127 ± 0.059 at 72 hr. There was no significant difference in the cell viability measurements of pSC exposed to BSA at any of these time points. Furthermore, we assessed the apoptotic effect of PA overload on AKT. AKT is a serine/threonine protein kinase and its activation plays a pivotal role in cell survival. To explore the potential effect of PA on the AKT pathway, we evaluated the effect of PA on AKT phosphorylation at Ser473 and Thr308 in pSC. As shown in Figure 1b, PA overload inhibits AKTp‐Ser473 and AKTp‐Thr308 in a time‐dependent manner. Of note, PA treatment did not affect total levels of AKT protein (Figure 1b).

**Figure 2:**
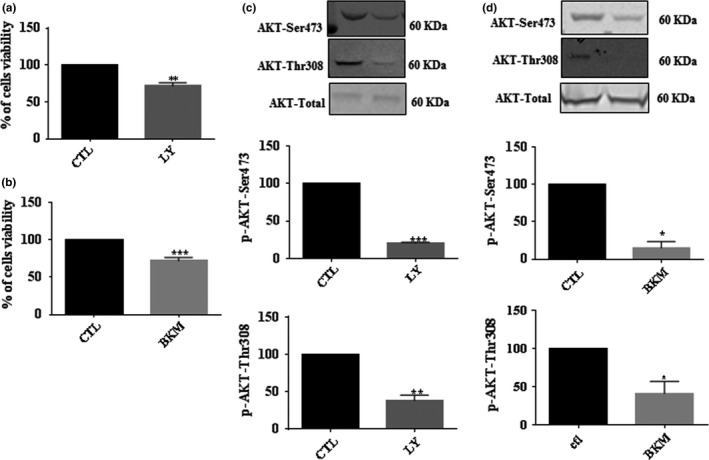
The Effect of PI3K/AKT inhibitors on primary Schwann cells (pSC) viability. pSC cultures were treated with PI3K inhibitors LY290042 (a), and BKM120 (b) for 48 hr followed by crystal violet assay to measure cell viability. The effect of LY290042 (c) and BKM120 (d) on AKT phosphorylation was examined by Western blot. The pSC cells lysates were prepared and subjected to specific antibodies against AKTp‐Ser473, AKTp‐Thr308, and total AKT protein. The blots were then analyzed using the Li‐Cor Odyssey system. A representative Western blot is shown above each bar graph. The data represent mean ± *SE* of at least four independent experiments. *p < 0.05, **p < 0.01, ***p < 0.001 vs. CTL

In the next series of experiments, we used selected inhibitors of PI3K, an upstream regulator of the AKT pathway to mimic the decrease in AKT phosphorylation by PA treatment. As expected, LY290042 and BKM120 decreased AKT phosphorylation at the Ser473 and Thr308 residues in pSC (Figure 2). Furthermore, LY290042 and BKM120 also decreased cell viability, suggesting a role of the PI3K/AKT pathway for Schwann cell survival.

### 3.2 DHA reverses the apoptotic cell death and the inhibition of AKT phosphorylation caused by PA‐LTx in primary cultured Schwann cells

**Figure 3:**
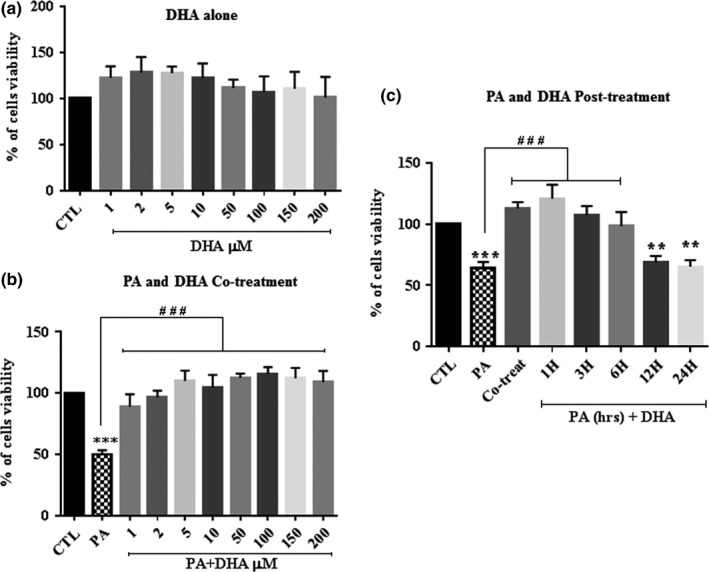
The Effect of co‐treatment and post‐treatment of DHA on primary cultured Schwann cells (pSC) viability under PA‐LTx. pSC cultures were treated with BSA alone (CTL), with PA:BSA (2:1) alone, with DHA (50–200 µM) alone (a) or co‐treated with PA:BSA (2:1) and DHA (50–200 µM) (b) for 48 hr and then cell viability was measured by crystal violet assay to measure cell viability. Alternately, the cells were post‐treated with DHA (50 µM) at 1, 3, 6, 12, or 24 hr after an initial PA exposure, and cell viability was measured at 48 hr (c). The data represent mean ± *SEM* of at least four independent experiments. **p < 0.01 and ***p < 0.001 are shown above the bars when compared to control groups. **^###^** *p* < 0.001 are shown above the lines when compared between two linked groups

**Figure 4:**
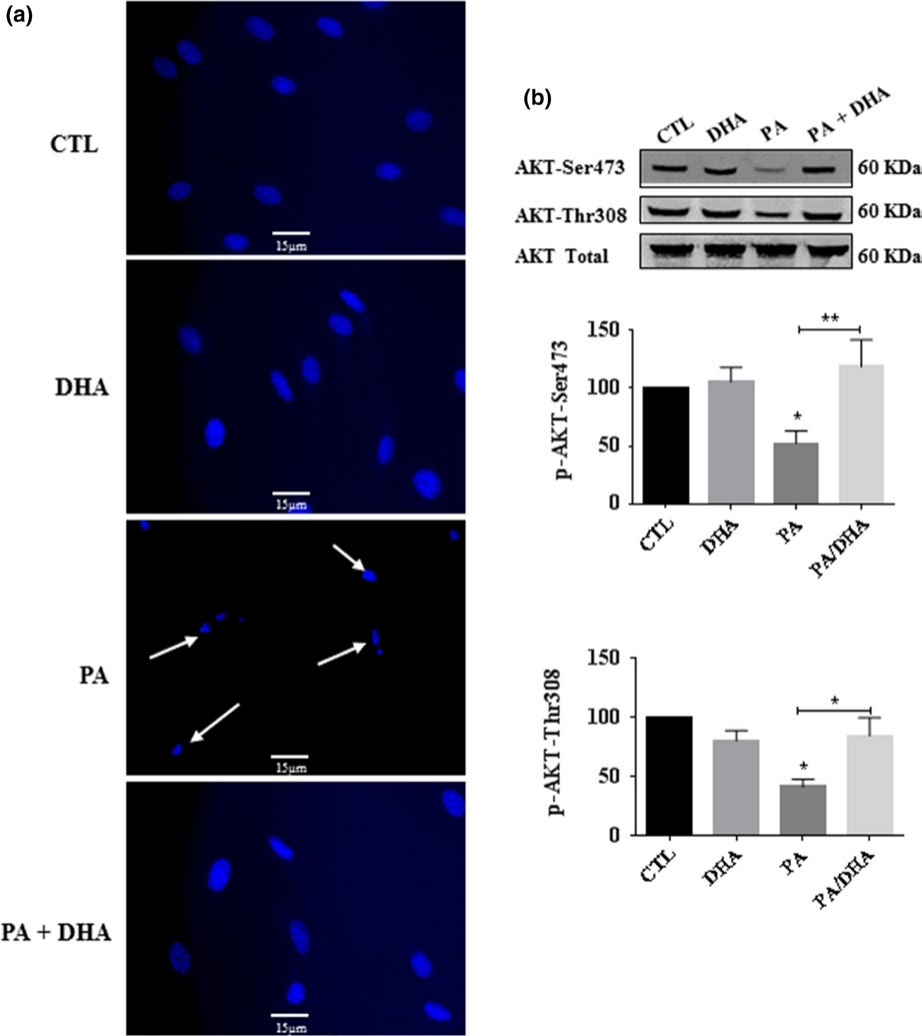
DHA eliminates apoptotic cell death and restores AKT phosphorylation in primary cultured Schwann cells (pSC) under PA‐LTx. pSC were treated with BSA alone (CTL), with PA:BSA (2:1) alone, with DHA (50 µM) alone or co‐treated with PA:BSA (2:1) and DHA (50 µM) for 48 hr. Nuclear morphology was determined by Hoechst staining. Nuclear condensations are indicated with white arrows (a). The pSC culture lysates were prepared and subjected to Western blot analysis using specific antibodies against AKTp‐Ser473, AKTp‐Thr308, and total AKT protein. The blots were then analyzed using the Li‐Cor Odyssey system. A representative Western blot is shown by each bar graph (b). The data represent mean ± *SEM* of at least four independent experiments. *p < 0.05 are shown above the bars when compared to control groups. **^#^** *p* < 0.05 and **^##^** *p* < 0.01 are shown above lines when compared between two linked groups

In a previous report, we show that DHA protects nerve growth factor‐differentiated pheochromocytoma cells and neuronal cortical cells against PA‐LTx (Almaguel et al., 2009). The next series of experiments assessed DHA treatment on the survival of Schwann cells undergoing PA‐induced lipotoxicity. Our findings show that co‐treatment of PA with DHA completely reversed the cell death induced by PA‐LTx (Figure 3b), whereas DHA 1 µM to 200 µM (DHA:BSA, 1:150 to 4:3) alone had no effect on Schwann cell viability (Figure 3a), showing that the wide range of DHA concentration used in these experiments was not toxic for these cells. We observed that DHA conferred protection in all the concentrations tested (1–200 µM; Figure 3b). Next, we examined the minimum PA exposure time required to produce irreversible lipotoxicity by treating pSC cultures with DHA after the addition of PA (post‐treatment; Figure 3c). Treating the cells with DHA (50 µM) at 1, 3, or 6 hr after an initial PA exposure resulted in significant protection. However, the protective effect of DHA was not observed in pSC exposed to PA for 12 and 24 hr (Figure 3c). These results suggest that the PA‐induced cell death cascade is fully reversible when DHA is added within the first 6 hr. We confirmed the anti‐apoptotic effects of DHA by Hoechst staining (Figure 4a). The nuclear morphological micrographs show that the cells exposed to PA exhibit nuclear condensation and apoptotic bodies. However, co‐treatment with DHA resulted in pSC showing normally dispersed chromatin and an intact nuclear membrane as in the control cells. DHA (50 µM), (DHA:BSA, 1:3), alone had no effect on the morphology and nuclear integrity of the cells. Similarly, on human skeletal muscle cells, studies showed that the use of 10–400 µM of DHA conjugated with 2% BSA or a ratio DHA:BSA 5:1, conditions similar to ours, had no deleterious effects on these cells (Lam et al., 2011; Sawada et al., 2012).

**Figure 5:**
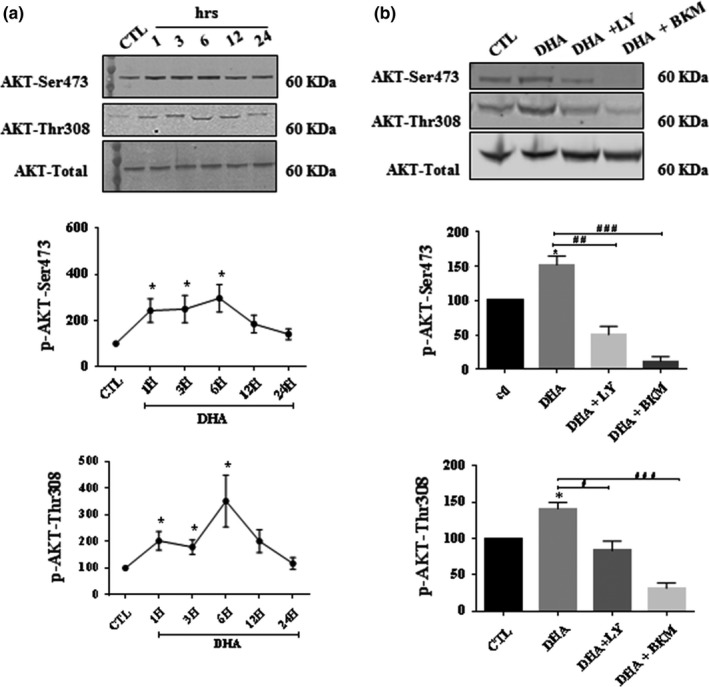
The increase in AKT phosphorylation in primary cultured Schwann cells (pSC) by DHA is abolished by PI3K inhibitors. pSC were treated with DHA (50 µM) for (1–24 hr) (a). pSC were treated with DHA in the presence or absence of LY294002(40 µM), BKM120 (2 µM) and for 6 hrs (b). pSC cell lysates were prepared and subjected to Western blot analysis using specific antibodies against AKTp‐Ser473, AKTp‐Thr308, and total AKT. The blots were then analyzed using the Li‐Cor Odyssey system. The data represent mean ± *SEM* of at least three independent experiments. A representative Western blot is shown above each bar graph. *p < 0.05, when compared to the control groups. **^#^** *p* < 0.05 and **^###^** *p* < 0.01 are shown above lines when compared between two linked groups

The next series of experiments evaluated the effect of DHA on AKT phosphorylation at Ser473 and Thr308 in pSC overloaded with PA. We found that PA decreased AKTp‐Ser473 and Thr308. However, in the presence of DHA, we observed that the p‐AKT levels were not different than control (Figure 4b). The pSC cultures treated with DHA alone showed a transient increase in phosphorylation of AKT‐Ser473 and Thr308 at 1, 3, and 6 hr (Figure 5a). However, when we added LY294002 or BKM120, the induction of AKT phosphorylation caused by DHA decreased (Figure 5b). Altogether, the data indicate that DHA may rescue Schwann cells from PA‐LTx through the AKT pathway.

### 3.3 Implication of PI3K/AKT pathway on DHA protective effects

**Figure 6:**
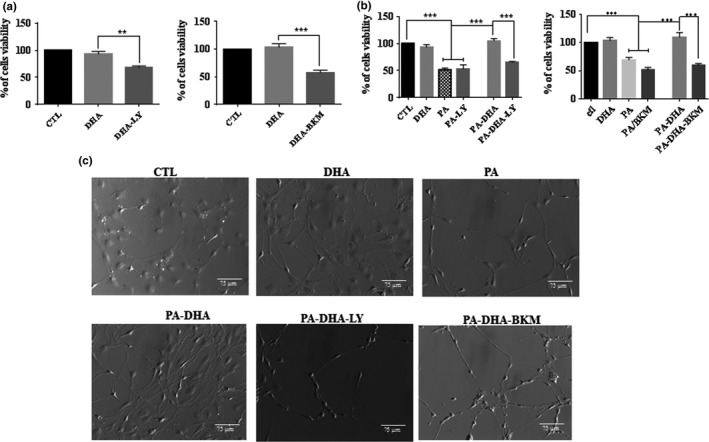
The protective effect of DHA on primary cultured Schwann cells (pSC) under PA‐LTx is abolished by PI3K/AKT inhibitors. pSC cultures were treated with DHA (50 µM) in the presence or absence of PI3K inhibitors LY290042 (40 µM) or BKM120 (2 µM) (a). The pSC cultures were treated with PA:BSA (2:1), PA + DHA in a presence or absence of PI3K inhibitors LY290042 (40 µM), or BKM120 (2 µM) (b). Cell viability was measured at 48 hr by crystal violet assay. Cells morphology was visualized at 48 hr by Hoffman modulation contrast microscopy. Membrane blebs and cell shrinkage are indicated with white arrows (c). The data represent mean ± *SEM* of at least five independent experiments **p < 0.05, ***p < 0.001 when compared between two linked groups

To further investigate the implication of PI3K/AKT signaling in the protective effect of DHA, we evaluated the effect of DHA against PA‐LTx in pSC in the presence of the PI3K inhibitors LY294002 and BKM120. We found that LY294002 and BKM120 (Figure 6b) reversed the protective effect of DHA against PA‐LTx. Also, LY294002 and BKM120 decreased cell viability in the presence of DHA (Figure 6a). Since LY294002 and BKM120 alone decreased pSC cell viability (Figure 2), the PI3K/AKT pathway most likely plays a critical role in pSC cell survival and DHA may exert its protective effect through it. In addition, PA‐treated cells presented apoptotic features like membrane blebs and cell shrinkage, while co‐treatment with DHA prevented the apoptotic features induced by PA‐LTx. However, adding LY294002 and BKM120 to PA‐DHA co‐treated pSC elicited the apoptotic features seen in cells exposed to PA alone (Figure 6c).

### 3.4 Implication of mTOR pathway on DHA protective effects

**Figure 7:**
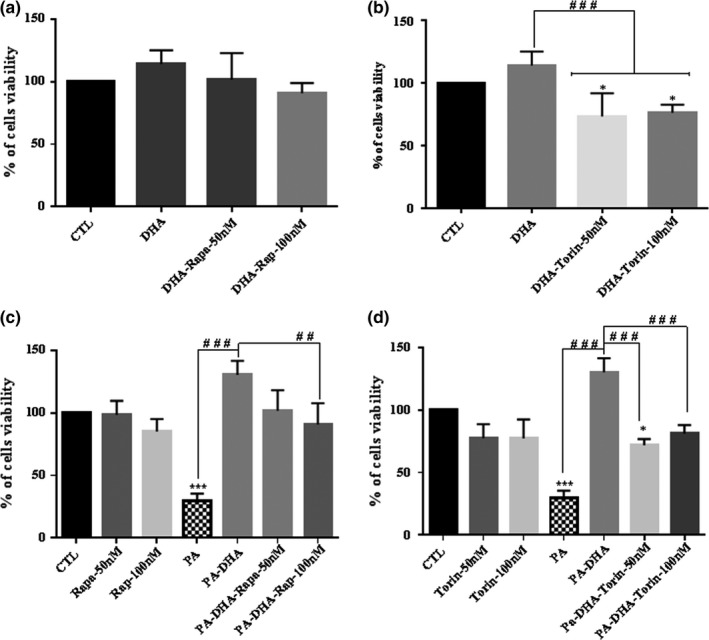
The protective effect of DHA on primary cultured Schwann cells (pSC) under PA‐LTx is partially inhibited by mTOR inhibitors. pSC cultures were treated with DHA (50 µM) in a presence or absence of mTOR inhibitors rapamycin (50 and 100 nM) or Torin 1 (50 and 100 nM) (a, b). The pSC cultures were treated with PA:BSA (2:1), PA + DHA in the presence or absence of mTOR inhibitors rapamycin (50 and 100 nM), or Torin 1 (50 and 100 nM) (c, d). Cell viability was measured at 48 hr by crystal violet assay. The data represent mean ± *SEM* of at least five independent experiments **^##^** *p* < 0.01, **^###^** *p* < 0.001 when compared between two linked groups and * *p* < 0.05, *** *p* < 0.001 when compared with the control

To explore a potential role of the mTOR pathway on DHA protection of Schwann cells under PA‐LTx, cells were treated with 50 and 100 nM of rapamycin or Torin‐1 for 48 hr. Rapamycin is a natural product that blocks mTORC1 (Igarashi & Satoh, 1989), while Torin 1 is a synthetic molecule that blocks mTORC1 and mTORC2 (Liu et al., 2010; Thoreen et al., 2009). The results showed that addition of rapamycin to PA‐DHA conditions partially inhibited the neuroprotective effects of DHA at 100 nM compared to the PA‐DHA group. However, there was no difference between PA‐DHA‐rapamycin groups and control (Figure7c). On the other hand, the addition of Torin 1 partially inhibited the protective effect of DHA against PA‐LTx at 50 and 100 nM. Furthermore, the presence of 50 nM of Torin 1 to PA‐DHA significantly decreased Schwann cell viability when compared to the control group (Figure 7d). In addition, Torin 1, but not rapamycin, decreased cell viability in the presence of DHA (Figure 7a,b). Taken together, these data suggest the implication of mTORC2 in DHA rescuing of Schwann cells from PA‐LTx.

## 4 DISCUSSION

Without proper intervention, Schwann cells exposed to PA overload exhibit dysfunction and may die through apoptosis. This PA‐LTx‐induced apoptotic cell death induces ER stress, ROS generation, and mitochondrial depolarization. For instance, BAPTA, a calcium chelator, and MC‐186, an antioxidant, fully block the cell death induced by PA‐LTx (Padilla et al., 2011). The present study shows that lipotoxicity is accompanied by a decrease in AKT phosphorylation. We also report that DHA reverses the dysfunction and cell death associated with PA‐LTx. Treatment with DHA stimulates AKT phosphorylation, and addition of PI3K inhibitors blocks the beneficial effects of DHA. These findings support the hypothesis that DHA mediates its protective effect at least in part through the stimulation of the PI3K/AKT and mTORC2 pathways.

AKT, a target of PI3 kinase activation, is a key signaling molecule that contributes to biological responses such as cell proliferation, cell survival, and apoptosis (Chaanine & Hajjar, 2011; Datta, Brunet, & Greenberg, 1999). Several studies have suggested the importance of the PI3K/AKT pathway in normal Schwann cell function (Colognato & Tzvetanova, 2011; Maurel & Salzer, 2000). AKT is important for Schwann cell migration after peripheral nerve injury (Yu et al., 2015); an overexpression of AKT is sufficient to inactivate Bad and reduce apoptosis after serum withdrawal (Li, Tennekoon, Birnbaum, Marchionni, & Rutkowski, 2001). The present study shows that palmitic acid overload decreases cell viability and induces apoptosis in Schwann cells. This induction of apoptosis is accompanied by a decrease in AKT phosphorylation at Ser473 and Thr308. PA overload has also been shown to induce apoptosis by decreasing AKT phosphorylation in other cells like islet endothelial cells, cardiomyocytes, and pancreatic β‐cells (Chen et al., 2013; Wei et al., 2012; Yuan, Lu, Wang, Li, & Li, 2014), but the mechanism is not well understood. Chen et al showed that PA induced downregulation of p‐AKT‐Ser 473 and was reversed by TUDCA, an ER stress inhibitor (Chen et al., 2013). Recently, we reported that PA induced ER stress in immortalized Schwann cells (Padilla et al., 2011). This earlier response was associated with a calcium decrease in the ER lumen at 15 min and it was followed by an increase in GRP78 and CHOP, two well‐known markers of ER stress. We reported that when the cytosolic calcium levels were reduced by BAPTA, the levels of GRP78 and CHOP were restored to the control levels, which in turn rescued the cells from PA‐Ltx (Padilla et al., 2011). Furthermore, few studies have demonstrated that PA mediates its lipotoxic effects through the production of lipid metabolites such as diacyl glycerol, acyl carnitines, acyl‐CoA, and ceramides (Capel et al., 2015; Chavez et al., 2003; Schmitz‐Peiffer, 2000). These molecules mediate the activation of protein kinase C‐θ and pro‐inflammatory signals. In skeletal muscle cells, Capel et al showed that PA induced production of ceramide, which is associated with a decreased of AKT phosphorylation under insulin stimulation (Capel et al., 2015). However, the presence of myriocin, a ceramide inhibitor, partially restored the activity of AKT (Capel et al., 2015).

An important finding of the present study is that DHA rescues Schwann cells from apoptotic cell death triggered by PA‐LTx. There is a gap in the literature about the mechanism (s) by which DHA confers cell protection against lipotoxicity. We show that DHA rescues SC from PA‐LTx and that this DHA protection involves the restoration of the AKT phosphorylation of Ser473 and Thr308 trending toward control levels. In addition, in the presence of PI3K inhibitors, such as LY294002 and BKM120, DHA was no longer able to restore phosphorylation of AKT and had no effect on the SC survival under the PA‐LTx condition. Consistent with our findings, DHA has been shown to prevent the reduction in AKT phosphorylation at Ser473 and at Thr308 and prevents cell apoptosis induced by serum deprivation or by staurosporine treatment in neuro 2A cells (Kim, Akbar, & Kim, 2010; Kim, Akbar, Lau, & Edsall, 2000; Kim, Bigelow, & Kevala, 2004). In addition, the positive effects of DHA on AKT phosphorylation and on cell survival were inhibited in the presence of PI3K inhibitors (Kim et al., 2010, 2000, 2004). These observations, together with our findings, suggest that the DHA neuroprotective effect occurs by increasing AKT phosphorylation resulting in an activation of the PI3K pathway. This hypothesis is supported by the observation that DHA alone is able to increase AKT phosphorylation and that this increase was blocked by the PI3K inhibitors LY294002 and BKM120. In addition, we also show that the PI3K inhibitors, LY249002 and BKM120, decreased AKT phosphorylation resulting in cell death. Our data are consistent with a previous study showing that LY249002 increased the number of apoptotic Schwann cells (Li et al., 2001).

Schwann cells play a significant role in neuronal integrity (Sun et al., 2012). Given their essential functions in the peripheral nervous system and their vulnerability under metabolic changes that occur during diabetes, maintaining the function and the integrity of Schwann cells is important in the treatment of diabetic peripheral neuropathy (Sun et al., 2012; Suzuki et al., 2011). DHA has beneficial effects on peripheral neuropathy; a diet supplemented with fish oil or daily doses of DHA improved the internal diameter of myelinated fibers, Na, K‐ATPase activity, and nerve conduction velocity in the sciatic nerve of Streptozotocin‐induced (STZ) diabetic rats (Coste, Gerbi, Vague, Pieroni, & Raccah, 2003; Gerbi et al., 1999). In our study, we found that the protective effects of DHA were not observed if it was added 12 hr after exposure to PA. However, the effect was significant when the DHA was added within 6 hr after PA exposure, suggesting that the neuroprotective effects of DHA are time sensitive. This knowledge can have therapeutic applications and is consistent with previous findings showing a therapeutic value for DHA in inhibiting neuropathic pain during spinal cord injury. In these published studies, acute parenteral administration of DHA prior to injury and a diet rich in fish oil‐derived omega‐3 fatty acids increased the levels of AKT, increased the percentage of white matter sparing, resulted in axonal preservation, and improved the conduction and locomotor function after spinal cord injury (SCI; Figueroa et al., 2012, 2013 ).

The underlying mechanism by which DHA activates AKT is not well defined. Two steps are involved in AKT activation. First, AKT must be translocated from the cytoplasm to the membrane and then phosphorylated at the Thr308 and Ser475 sites (Alessi & Cohen, 1998; Downward, 1998). The activation of PI3K results in a transformation of 4,5‐phosphatidylinositol biphosphate (PIP2) to phosphatidylinositol 3,4,5‐triphosphate (PIP3) in the plasma membrane. PIP3 formation triggers AKT recruitment from the cytosol to the plasma membrane where it will be activated through phosphorylation by PDK1 at the Thr308 site and by mTORC2 at the Ser473 (Brazil & Hemmings, 2001; Cantley, 2002; Kim et al., 2017; Sancak et al., 2007; Sarbassov, Guertin, Ali, & Sabatini, 2005; Vander Haar et al., 2007). Kim et al., in their DHA neuronal enrichment model, showed that DHA is incorporated into neuronal membrane phospholipids mainly into phosphatidyl serine (PS) to exert its effect by facilitating the translocation of AKT at the plasma membrane to be activated (Kim et al., 2010, 2000, 2004). Furthermore, DHA can be cleaved from the phospholipid membrane by the action of phospholipase A_2_; free DHA is then oxygenated by 15‐lipoxygenase‐1 (15‐LOX‐1) to form neuroprotectin D1 (NPD1), a metabolite of DHA (Bazan, 2009). NPD1 can mediate its actions in an autocrine or paracrine manner (Bazan, 2009). NPD1 may have neuroprotective effects during stoke and in retinal pigment epithelial cells: NPD1 decreased apoptotic cell death, increased AKT phosphorylation and mTOR under oxidative stress conditions (Halapin & Bazan, 2010; Mukherjee, Marcheselli, Serhan, & Bazan, 2004). In this present study, our data suggest that mTORC2 may be implicated in DHA protection of Schwann cells against PA‐LTx by regulating AKT. Yao et al found that mSin1 a unique component within mTORC2 was associated with AKT. When the AKT binding site was deleted from mSin1, AKT phosphorylation at Ser473 was greatly decreased. Furthermore, the association between AKT and mTOR was disrupted by serum withdrawal and addition of LY294002 but not by rapamycin, suggesting that it may be a result of the dissociation of AKT from mTORC2 (Yao et al., 2017). Altogether these data suggest that PA‐LTx may dissociate AKT from the mTORC2 complex allowing decreased levels of AKT phosphorylation at Ser473, however, AKT phosphorylation is reinstated when DHA is present. In addition, NPD1 rescues cells from death by increasing the anti‐apoptotic proteins such as Bcl‐xL and Bcl‐2, downregulating pro‐apoptotic proteins like Bax and Bad (Mukherjee et al., 2007, 2004 ). In this regard, it is possible that DHA protects Schwann cells from PA‐LTx by inhibiting apoptosis and increasing AKT phosphorylation through NPD1.

In conclusion, we report that PA decreased the phosphorylation of AKT and induced apoptotic cell death in Schwann cells. DHA protects Schwann cells from PA‐LTx and can also reverse the PA‐LTx insult on Schwann cells. This effect is mediated, at least in part, through the PI3K/AKT and mTORC2 pathways. Given the important function of Schwann cells in the peripheral nervous system, understanding the mechanisms by which DHA maintains Schwann cell function and adds to their integrity could have a key role in the treatment of diabetic peripheral neuropathy.

## CONFLICT OF INTEREST

The authors declare no conflict of interest.

## AUTHOR CONTRIBUTIONS

All authors had full access to all the data in the study and take responsibility for the integrity of the data and the accuracy of the data analysis. *Conceptualization:* M.D. and M.D.L.; *Methodology:* M.D.L., M.D.; *Validation:* K.F. and M.S.I.; *Investigation:* M.D.; *Formal analysis*: M.D.L. and M.D.; *Resources*: M.D.L.; *Data Curation:* M.D. and M.D.L; *Writing‐Original draft*: M.D.; *Writing‐Review and Editing*: M.D. and M.D.L.; *Visualization*: M.D.; *Supervision:* M.D.L.
